# The Double Within: A review of the phenomenology and psychopathology of Autoscopic Phenomena

**DOI:** 10.1192/j.eurpsy.2025.2044

**Published:** 2025-08-26

**Authors:** A. F. Reis, T. Cardoso, C. P. Ramos, M. M. Figueiredo, J. Marta, M. J. Freire

**Affiliations:** 1 Psychiatry and Mental Health, Unidade Local de Saúde da Arrábida, Setúbal, Portugal

## Abstract

**Introduction:**

Autoscopic Phenomena (APs) are rare perceptual experiences where individuals perceive a visual double or duplicate of their own body. It has been recognized since ancient times, but gained significant attention in the 19th century, both through its depiction in romantic literature and in neuropsychiatric studies; in the literature, their association with epilepsy and schizophrenia is very extensively documented. Recent research brought new insights into the neurobiology of these phenomena.

**Objectives:**

In this article, we aim to review the phenomenology and psycopathology of APs.

**Methods:**

Narrative literature review.

**Results:**

From a phenomenological perspective, three main conditions can be identified. In Autoscopy, the person sees a double, but does not feel a connection with it, meaning that they can distinguish between themselves and the double. In Out-of-body Experience (OBE), the individual feels as though they have left their body and observe themselves from an external perspective. In Heautoscopy, the boundary between the self and the double is blurred, causing uncertainty about where one’s “self” is located, representing a middle ground between autoscopy and OBE.

Current research suggests that APs are linked to dysfunctions in the normal integration of body ownership, self-location, and perspective-taking, caused by lesions in regions responsible for integrating multisensory inputs (visual, proprioceptive and vestibular). All types of APs have in common a dysfunction specifically in the temporo-parietal junction (TPJ), a brain area involved in processing self-location and integrating sensory inputs to create a unified sense of self; other common areas include the insula and cingulate cortex.

Regarding to the different networks of APs, Autoscopy primarily involves abnormalities in the visual processing regions (as the occipital and parietal lobes), causing a visual perception of double; OBEs are caused by dysfunctions in areas responsible for self-location and vestibular processing (such as the medial prefrontal cortex), leading to a sensation of floating outside one’s body; lastly, Heautoscopy engages more widespread brain dysfunctions, including the regions involved in self-representation and embodiment, leading to ambiguity in self-location.

**Conclusions:**

APs challenge our understanding of the bodily self and how identity is constructed, raising questions about how the brain creates a unified sense of being in a body and how this can break down under certain pathological conditions. Although much is unknown, one thing is for sure: these phenomena demonstrate that the sense of self is not fixed, and the study of its disruption, by exploring its phenomenology and psychopatology, may contribute to reveal the underlying processes involved in bodily self-consciousness.

**Disclosure of Interest:**

None Declared

